# Supplementary material for: Elevated Procalcitonin Is Positively Associated with the Severity of COVID-19: A Meta-Analysis Based on 10 Cohort Studies
Source: Medicina (Kaunas). 2021 Jun 9;57(6):594. doi: 10.3390/medicina57060594 (PMC8227321; doi:10.3390/medicina57060594)
Supplement: Supplementary file 1 [file medicina-57-00594-s001.zip › medicina-1213842-supplementary.pdf]

Elevated procalcitonin is positively associated with the severity of COVID-19: A meta-analysis based on 10 cohort studies

SUPPLEMENTARY FILE.

Figures & Tables

Table S1 Search terms

**PubMed**	(Procalcitonin OR PCT) AND (COVID-19 [Supplementary Concept] OR "severe acute respiratory syndrome coronavirus 2" [Supplementary Concept] OR SARS-CoV-2 OR "severe acute respiratory syndrome coronavirus 2" OR "novel coronavirus 2019" OR 2019-nCoV OR "coronavirus disease 2019" OR "novel coronavirus pneumonia")
**Web of science**	TS = ('COVID-19' OR severe acute respiratory syndrome coronavirus 2' OR SARS-CoV-2' OR novel coronavirus 2019' OR 2019 nCoV' OR 'coronavirus disease 2019' OR novel coronavirus pneumonia')<br>TS = ('Procalcitonin' OR 'PCT')
**Embase**	**COVID-19**<br>'COVID-19': ti, ab OR 'severe acute respiratory syndrome coronavirus 2': ti, ab OR 'SARS-CoV-2': ti, ab OR 'novel coronavirus 2019': ti, ab OR '2019 nCoV': ti, ab OR 'coronavirus disease 2019': ti, ab OR 'novel coronavirus pneumonia': ti, ab<br>**PCT**<br>'Procalcitonin' : ti, ab OR 'PCT' : ti, ab

Table S2 Assessment of the risk of bias in each cohort study with the Newcastle-Ottawa scale in meta-analysis

**Author**	**Year**	**Selection**				**Comparability**	**Outcome**			**Score**
		**1 Is the definition adequate?**	**2 Representativeness of the cases**	**3 Selection of controls**	**4 Outcome of interest**	**5 Comparability of cohorts**	**6 Assessment of outcome**	**7 Length of follow-up**	**8 Adequacy of follow-up**	
Chen T	2020	1	0	1	1	1	1	1	1	7
Bahl A	2020	1	1	1	1	1	1	1	1	8
Nicholson CJ	2020	1	1	0	1	1	1	1	1	7
Zhao Z	2020	1	1	1	1	1	1	1	1	8
Chen R	2020	1	1	1	1	1	1	1	1	8
Su W	2020	1	1	1	0	1	1	1	1	7
Hou H	2020	1	1	0	1	1	1	0	1	6
Muhammad R	2021	0	1	1	1	1	1	1	1	7
Montrucchio G	2021	1	1	1	0	1	1	1	0	6
Keski H	2021	1	1	1	1	1	1	1	1	8
Jiang M	2021	1	1	1	0	1	1	1	1	7

Abbreviations: "1" indicates that the study has adequately defined exposure and "0" indications the opposite

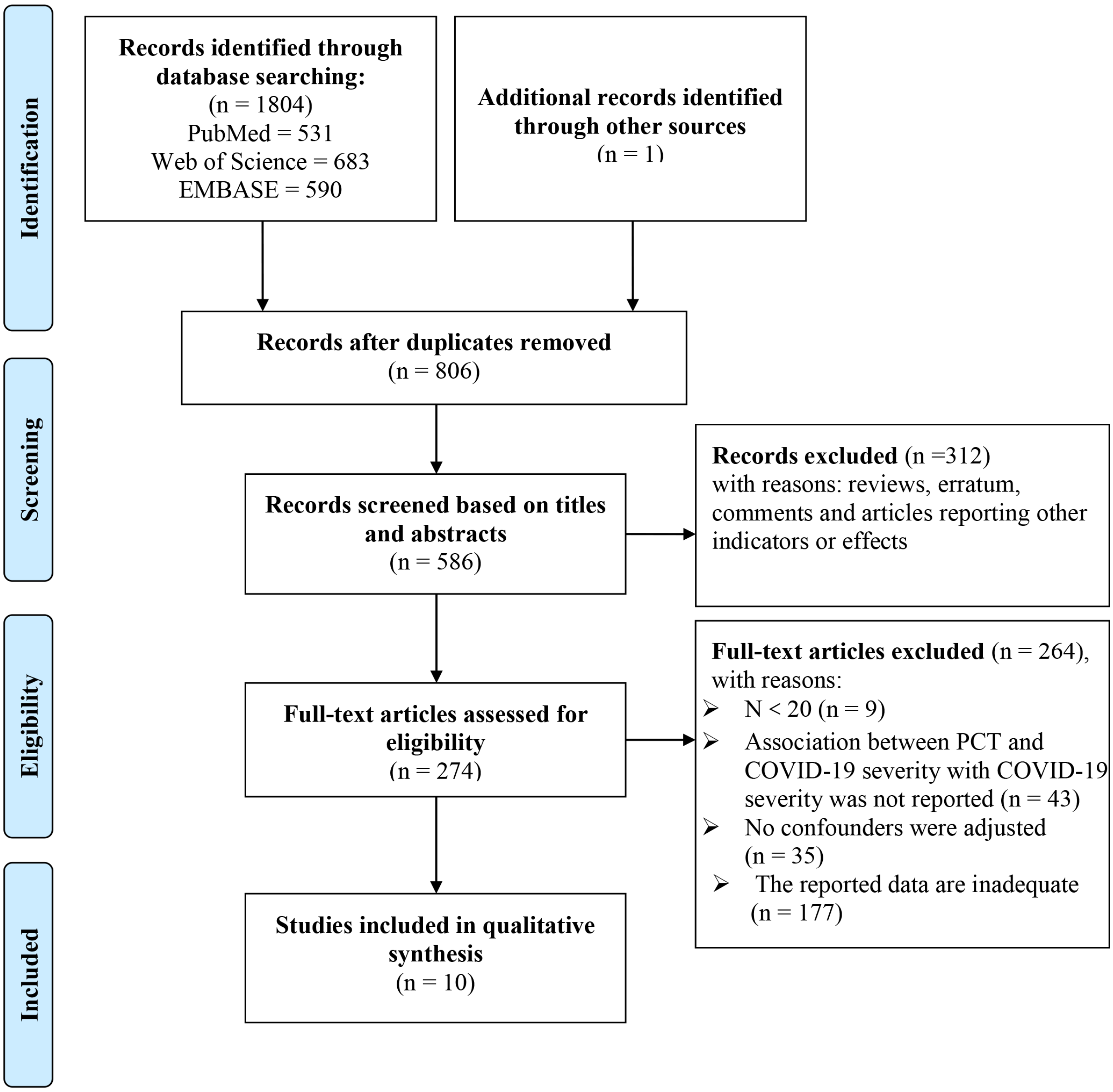

Figure S1 Flow diagram of study selection

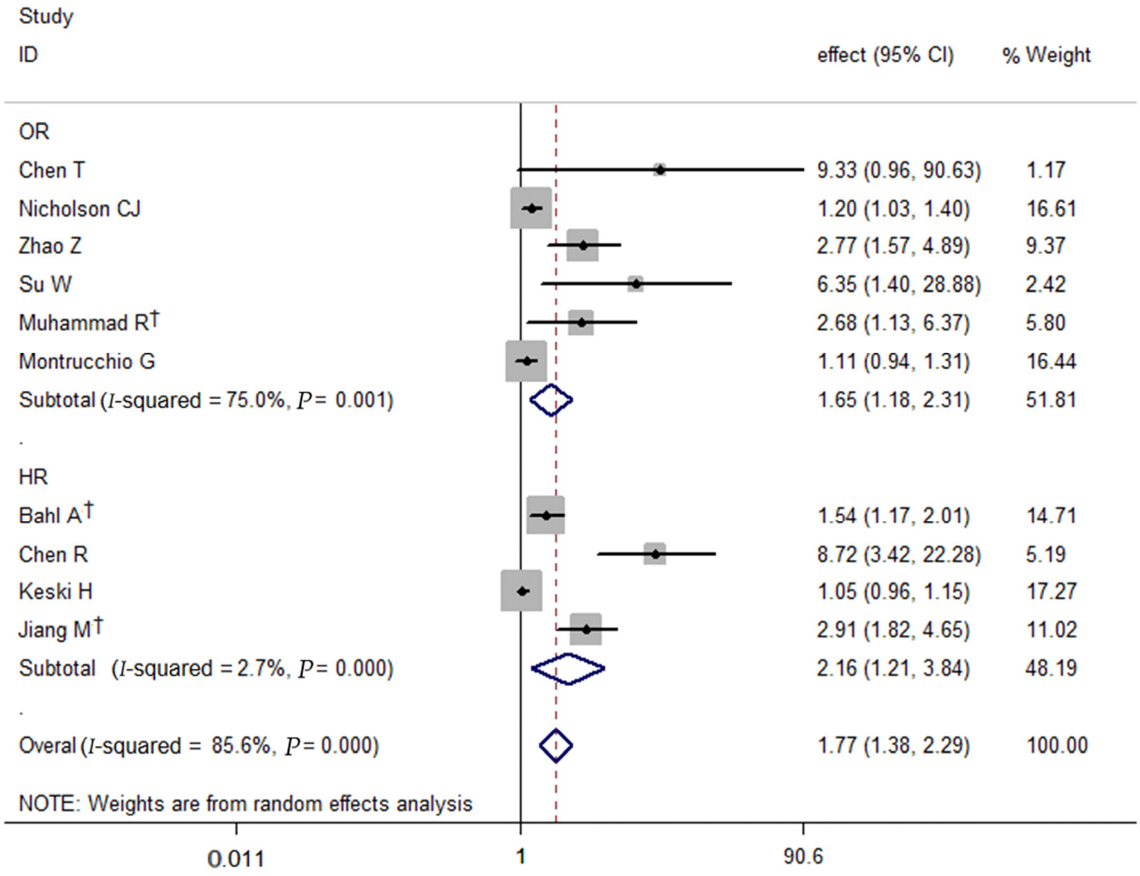

Figure S2 Subgroup analysis based on effect estimates for the association between PCT and the severity of COVID-19. † indicates combined effects based on subgroups

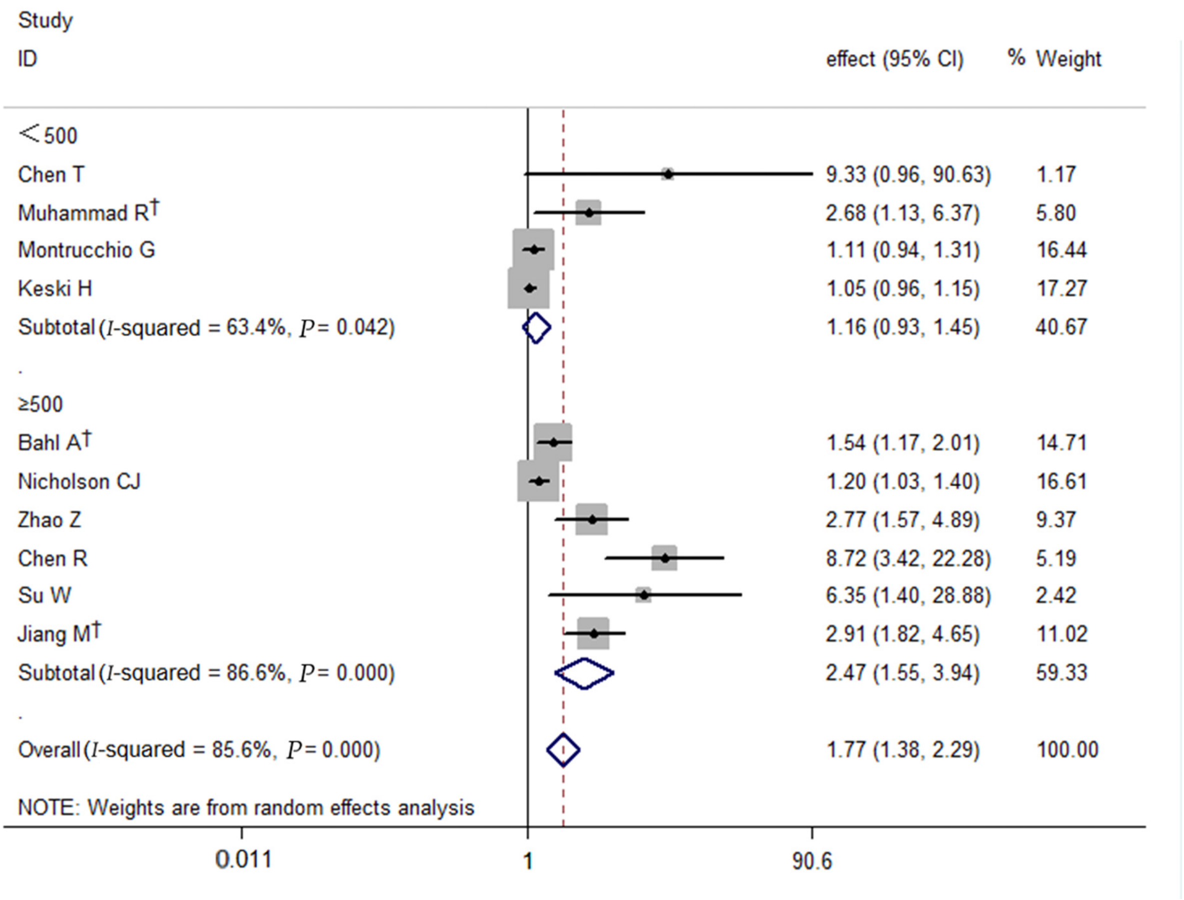

Figure S3 Subgroup analysis based on sample size for the association between PCT and the severity of COVID-19. † indicates combined effects based on subgroups

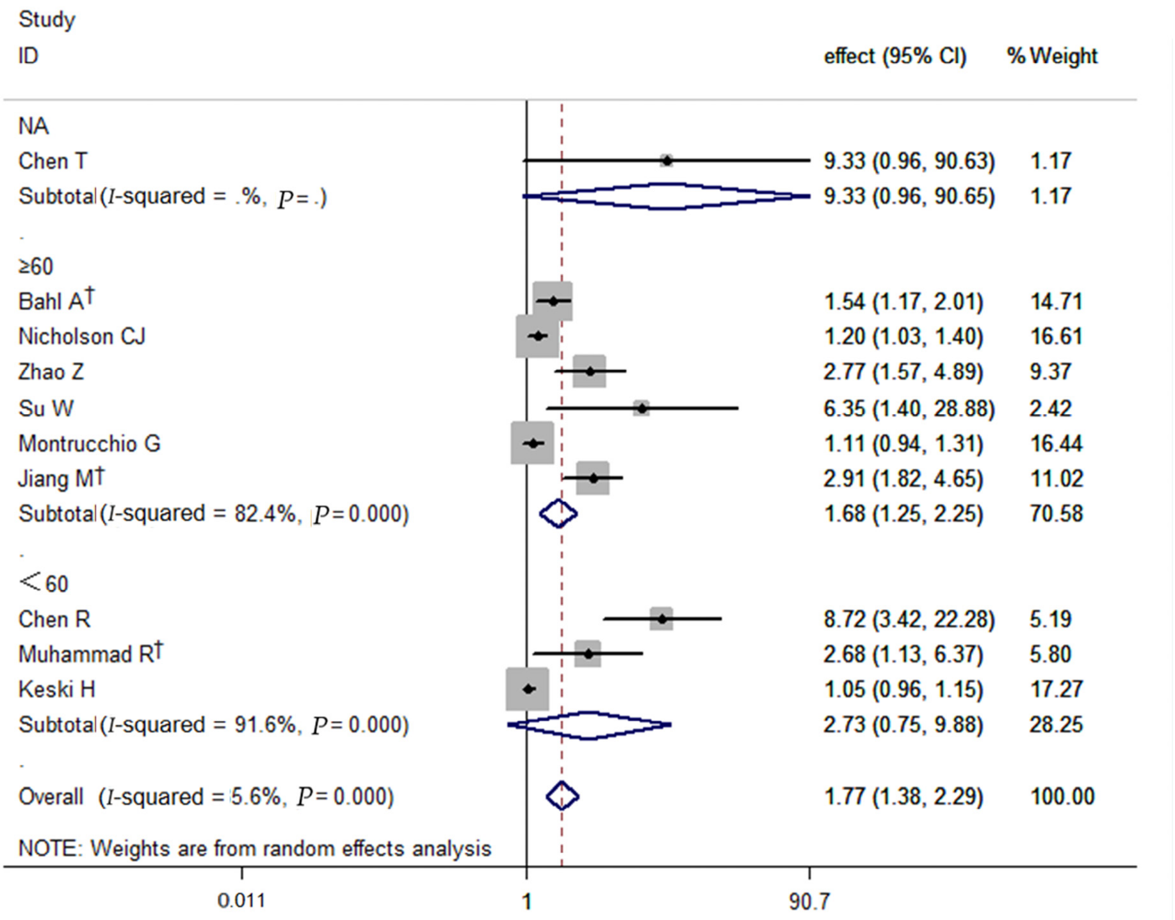

Figure S4 Subgroup analysis based on age for the association between PCT and the severity of COVID-19. NR, not reported. † indicates combined effects based on subgroups

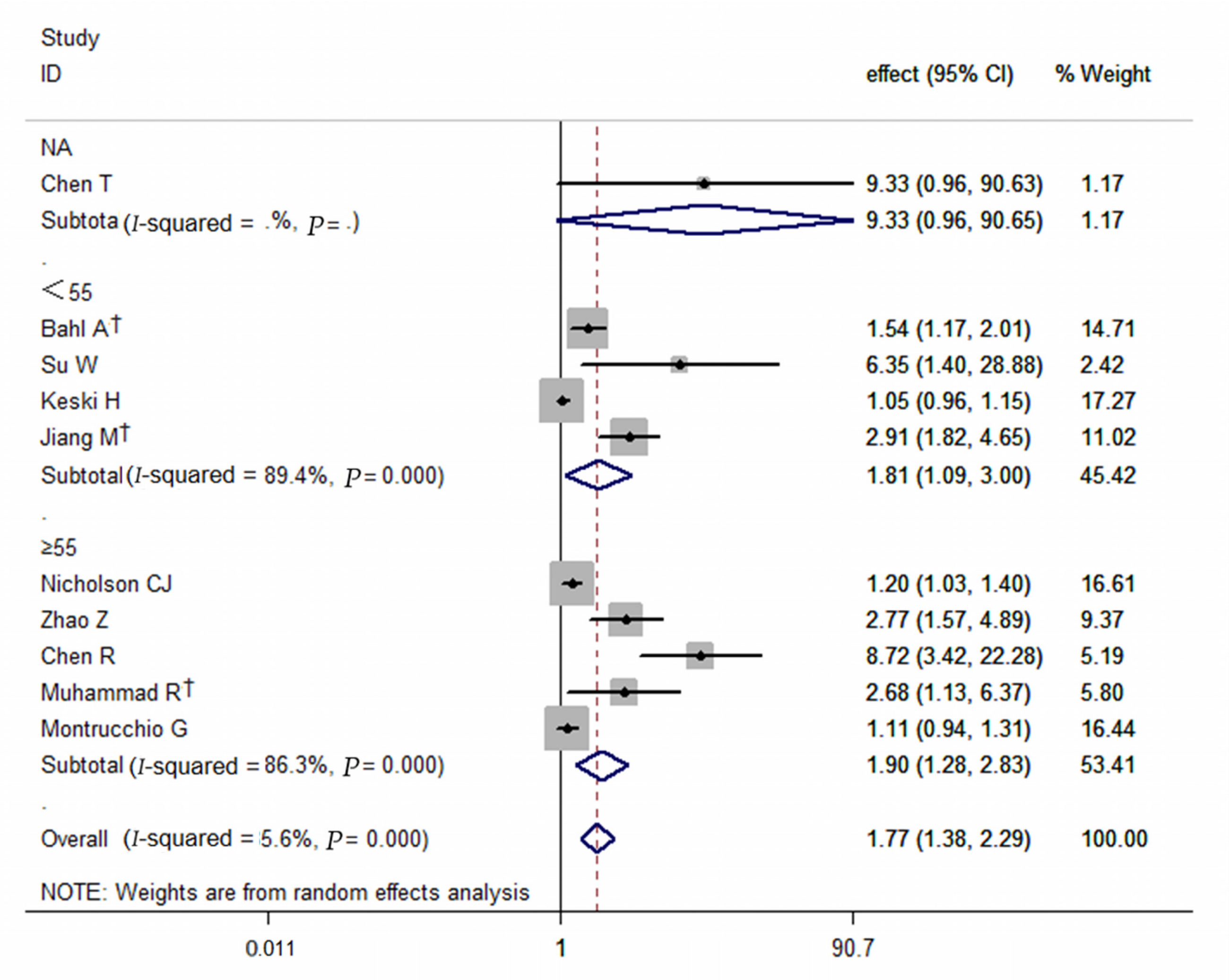

Figure S5 Subgroup analysis based on sex (male, %) for the association between PCT and the severity of COVID-19. NR, not reported. † indicates combined effects based on subgroups

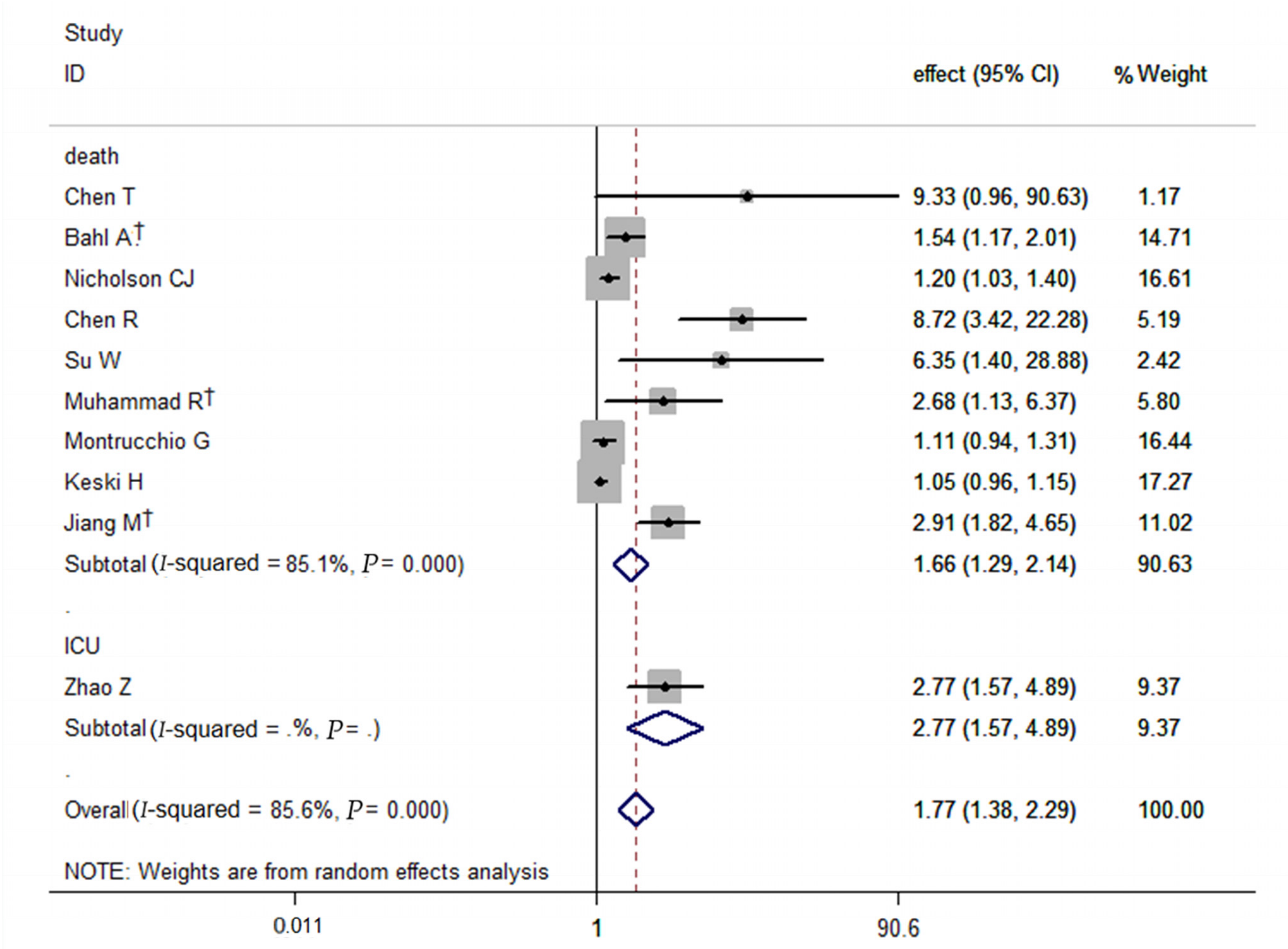

Figure S6 Subgroup analysis based on outcome for the association between PCT and the severity of COVID-19. Others include severe, critical, mechanically ventilated, and ICU patients. † indicates combined effects based on subgroups

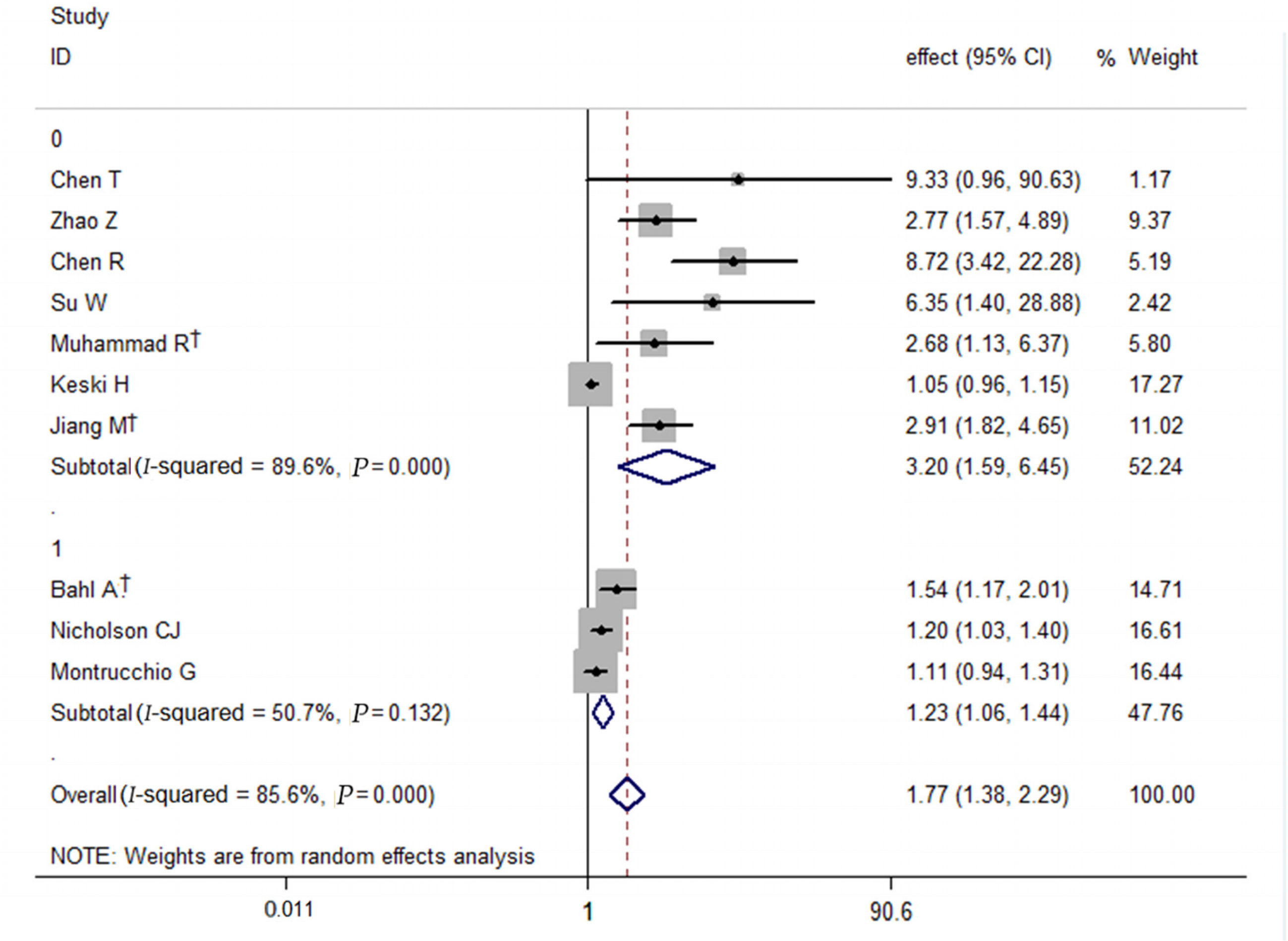

Figure S7 Subgroup analysis based on diabetes for the association between PCT and the severity of COVID-19. "1" indicates the study adjusted for diabetes. "0" indicates the study unadjusted for diabetes. † indicates combined effects based on subgroups

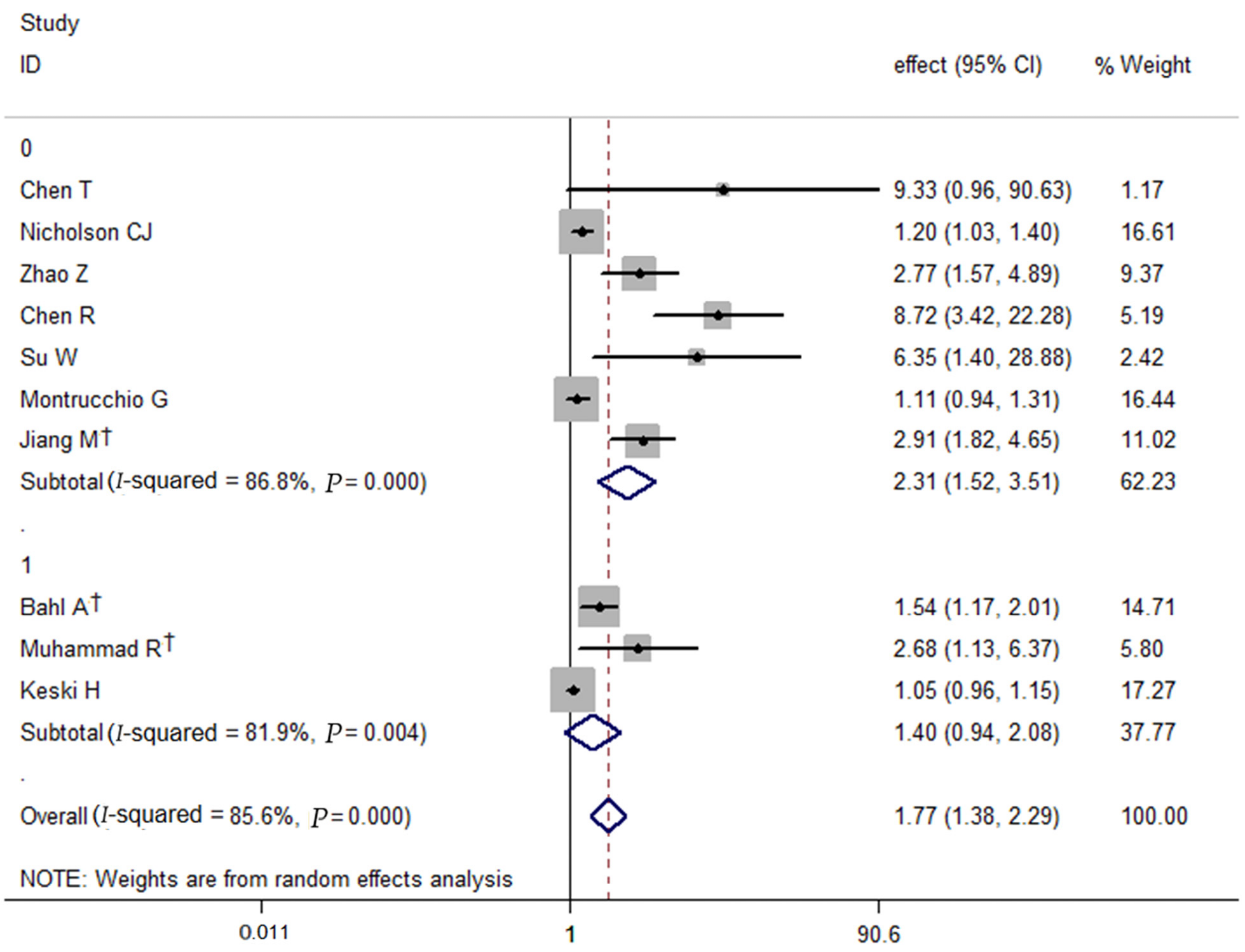

Figure S8 Subgroup analysis based on hypertension for the association between PCT and the severity of COVID-19. “1” indicates the study adjusted for hypertension. “0” indicates the study unadjusted for hypertension. † indicates combined effects based on subgroups
